# SERS-Based Immunoassays for the Detection of Botulinum Toxins A and B Using Magnetic Beads

**DOI:** 10.3390/s19194081

**Published:** 2019-09-21

**Authors:** Kihyun Kim, Namhyun Choi, Jun Ho Jeon, Gi-eun Rhie, Jaebum Choo

**Affiliations:** 1 Department of Chemistry, Chung-Ang University, Seoul 06974, Korea; sadtiger92@naver.com; 2 Department of Bionano Technology, Hanyang University, Ansan 426-791, Korea; choi.namhyun@gmail.com; 3 Division of High-risk Pathogens, Laboratory Control of Infectious Diseases, Korea Centers for Disease Control and Prevention, Chungju 28159, Korea; jhjeon78@korea.kr (J.H.J.); gerhie@korea.kr (G.-e.R.)

**Keywords:** surface-enhanced Raman scattering (SERS), botulinum neurotoxins, bioterrorism

## Abstract

Rapid and sensitive detection of botulinum neurotoxins (BoNTs) is important for immediate treatment with proper antitoxins. However, it is difficult to detect BoNTs at the acute phase of infection, owing to its rarity and ambiguous symptoms. To resolve this problem, we developed a surface-enhanced Raman scattering (SERS)-based immunoassay technique for the rapid and sensitive detection of BoNTs. Magnetic beads and SERS nanotags as capture substrates and detection probes, respectively, and Nile Blue A (NBA) and malachite green isothiocyanate (MGITC) as Raman reporter molecules were used for the detection of two different types of BoNTs (types A and B), respectively. The corresponding limits of detection (LODs) were determined as 5.7 ng/mL (type A) and 1.3 ng/mL (type B). Total assay time, including that for immunoreaction, washing, and detection, was less than 2 h.

## 1. Introduction

Botulinum neurotoxins (BoNTs) are regarded as one of the most serious high-risk biological agents used in bioterrorism. Considering their high toxicity (LD_50_∼1 ng/kg), ease of handling, and low price, BoNTs are listed as “Category A” bio-threat agents together with anthrax, plague, smallpox, tularemia, and viral hemorrhagic fevers by the US Centers for Disease Control and Prevention (CDCP) [1,2,3]. BoNTs have also been designated as lethal infectious agents for bio-terror by the Korea Centers for Disease Control and Prevention (KCDC). Seven different BoNT serotypes (serotype A–G) are known, of which the A, B, E, and F types are considered harmful to humans [4]. Intoxication with these BoNTs causes flaccid paralysis owing to the inhibition of neuromuscular signal transmission, which is identical for all serotypes. Muscle paralysis starts from the face, spreads to the whole body, and in severe cases, leads to death due to respiratory paralysis [5,6]. Immediate treatment with a specific antitoxin for a given toxin type is the only way to relieve the symptoms before the toxin enters nerve terminals [7,8]. Therefore, it is urgent to develop a rapid and accurate detection technique for toxic serotypes of BoNTs.

The gold standard method for the detection and identification of BoNTs is the mouse toxicity and neutralization bioassay (e.g., mouse bioassay), which is the only FDA-approved method to confirm the presence of active BoNTs [9,10]. However, this method has several drawbacks, including labor intensiveness, cost ineffectiveness, animal use, and time consumption (longer than four days). To resolve these problems, herein, we developed a surface-enhanced Raman scattering (SERS)-based immunoassay technique using magnetic beads [11,12,13,14,15]. The SERS-based bioassay technique has recently received great attention, owing to its high sensitivity and multiplex detection capability. Raman active sites called “hot junctions” show promise in their ability to overcome the low sensitivity problem associated with conventional Raman or fluorescence spectroscopy [16,17,18,19,20]. Herein, magnetic beads and gold nanoparticles (AuNPs) were used to capture antibody-supporting materials and detect antibody-conjugated sensing probes, respectively. This magnetic bead-based assay offers several advantages over conventional SERS assays using two-dimensional substrates [21,22,23]. First, the loading density of capture antibodies could be improved, as the three-dimensional magnetic beads have larger surface-to-volume ratio than any two-dimensional surface. Second, the assay does not require an extended incubation time because the fast molecular diffusion near three-dimensional beads facilitates faster kinetics of antibody–antigen assays. Finally, more reproducible detection is possible, as SERS signals are measured for the average ensembles of AuNPs in solution phase.

In the present study, the feasibility of our SERS-based magnetic immunoassay was investigated for the rapid and sensitive detection of BoNT types A and B. The limit of detection (LOD), sensitivity, and assay time were compared with those of an enzyme-linked immunosorbent assay (ELISA) for validation.

## 2. Materials and Methods

### 2.1. Materials

Gold (III) chloride trihydrate (HAuCl_4_·3H_2_O), tri-sodium citrate (Na_3_-citrate), 1-ethyl-3-(3-[dimethylamino]propyl) carbodiimide (EDC), N-hydroxysuccinimide (NHS), thiol-PEG-COOH (HS-PEG-COOH, MW∼3500), bovine serum albumin (BSA), Nile blue A (NBA), 3,3’,5,5’-tetramethylbenzidine (TMB), liquid substrate system for ELISA, horseradish peroxidase (HRP)-conjugated goat anti-rabbit polyclonal antibody, and HRP-conjugated goat anti-mouse polyclonal antibody were purchased from Sigma-Aldrich (St. Louis, MO, USA). Malachite green isothiocyanate (MGITC), phosphate-buffered saline (PBS) (10×, pH 7.4), and carboxylic acid-activated magnetic beads (Dynabeads MyOne^TM^) were obtained from Invitrogen (Eugene, OR, USA), whereas HRP conjugation kit (ab102890) was supplied by Abcam (Cambridge, UK). We procured 3,3’,5,5’-tetramethylbenzidine (TMB) buffer from GenDEPOT (Katy, TX, USA) and inactivated botulinum toxins A and B from the Korea Center for Disease Control and Prevention (KCDCP). Anti-botulinum toxin antibody sets were also provided by KCDCP. Commercial carboxylic acid-conjugated magnetic beads (Dynabeads^®^MyOne) were purchased from Thermo Fisher Scientific (Waltham, MA, USA). Ultrapure water (18 MΩ·cm^−1^) used in this work was obtained from Milli-Q water purification system (Billerica, MA, USA).

### 2.2. ELISA Test

Sandwich ELISA was performed to test the antibody-binding capability of BoNTs. For BoNT/A assay, monoclonal capture antibodies (100 μL, 0.5 μg/mL) in Na_2_CO_3_-NaHCO_3_ buffer (pH 9.6) were immobilized on the surface of a 96-well plate and incubated overnight at 4 °C. The wells were blocked with 200 μL of PBS buffer (containing 1% BSA) to reduce non-specific binding. After 2 h, BoNT/A (100 μL) in the range of 0–1 μg/mL was added to different wells and allowed to react for 2 h. Polyclonal detection antibodies and HRP-linked secondary antibodies were sequentially added at an interval of 2 h. After incubation for another 2 h, TMB solution was added to induce TMB-HRP enzymatic reaction. PBST containing 0.05% (v/v) Tween-20 (200 μL, thrice) was used for washing. TMB stop buffer was added to terminate the reaction and absorbance was measured at 450 nm wavelength. For BoNT/B assay, HRP-conjugated polyclonal detection antibodies were used instead of secondary antibodies to avoid any cross-reaction between capture antibodies and secondary antibodies. An HRP conjugation kit was used to attach HRP to detection polyclonal antibodies, as per the manufacturer’s instructions. Other steps were the same as those for BoNT/A.

### 2.3. Preparation of Antibody-Conjugated SERS Nanotags

AuNPs were prepared as previously reported with the seeded-growth method [24]. Briefly, 75 mL of 2.2 mM sodium citrate solution was heated to its boiling point under vigorous stirring and mixed with 0.5 mL of 25 mM chloroauric acid (HAuCl_4_) upon boiling. The change in the color of the solution from light yellow to bluish gray and then to soft pink was noted in 15 min. The resulting gold seed solution was cooled to 90 °C and sequentially treated with 0.5 mL of 60 mM sodium citrate and 0.5 mL of 25 mM HAuCl_4_ 12 times at an interval of 2 min. The color of the solution finally changed from pink to deep red. The solution was stirred for 30 min at 90 °C and cooled to room temperature. The shape and size distribution of AuNPs were characterized with dynamic light scattering (DLS) and transmission electron microscopy (TEM).

Antibody-conjugated SERS nanotags were also prepared, as previously reported. Two Raman reporters, 2.0 μL of 10^−5^ M MGITC and 1.5 μL of 10^−4^ M NBA, were added to 1.0 mL of AuNP solution and allowed to react for 30 min under constant stirring (500 rpm). In total, 40 μL of 12.5 μM HS-PEG-COOH linkers were added to each solution to facilitate their immobilization on the surface of AuNPs via Au-S bonds. After stirring for 3 h at room temperature, the PEGylated AuNPs were washed twice with DI water. To activate -COOH terminal groups on the surface of AuNPs, 4 μL of 0.5 mM EDC and 4 μL of 0.5 mM NHS were sequentially added. After 30 min, excess EDC/NHS was washed twice with DI water and the NHS-activated AuNPs were reacted with 30 μL of 0.1 mg/mL detection antibodies overnight at 4 °C. Approximately 100 μL of 1% (w/v) BSA aqueous solution was added to block any unbound sites on the surface of AuNPs. The mixture was shaken for additional 30 min and centrifuged at 5000 rcf for 10 min to remove unbound proteins. After discarding the supernatant, the pellets were re-dispersed in PBS buffer. 

### 2.4. Preparation of Antibody-Conjugated Magnetic Beads

The magnetic property of commercial beads is superparamagnetic, and the average diameter size is estimated to be 1 μm. Core material is composed of Fe_3_O_4_ and the surface was homogeneously coated with highly cross-linked polystyrene and hydrophilic layer of glycidyl ether. Their surface was conjugated with carboxylic acids for antibody immobilization. In brief, 200 μL of 0.5 mg/mL carboxylated magnetic beads were prepared using 15 mM MES buffer (pH 6). After washing thrice with MES buffer, the solution was incubated with 2.5 μL of 0.1 M EDC and 2.5 μL of 0.1 M NHS for 30 min, followed by washing of the beads thrice with MES buffer. The beads were treated with 5 μL of 1 mg/mL mouse anti-BoNT monoclonal antibody overnight at 4 °C with continuous shaking. After washing three times with PBS buffer, the reaction mixture was treated with 20 μL of 1% (w/v) BSA aqueous solution for 30 min at room temperature. Unreacted reagents were removed by washing the beads thrice with PBS. The final product was stored in PBS at 4 °C for further use.

### 2.5. SERS-based Immunoassay of BoNT/A and BoNT/B

Parallel sandwich immunoassays for BoNT/A and BoNT/B were performed with spiked samples at eight different concentrations. First, 40 μL of SERS nanotags and 20 μL of BoNT-spiked samples were mixed and allowed to react under constant stirring. After 30 min, 20 μL of antibody-conjugated magnetic beads were added and allowed to react for 1 h. The mixture was washed thrice with PBST and the immunocomplexes were resuspended in PBS and transferred to a capillary tube for Raman measurements.

### 2.6. Instrumentation

DLS measurement was performed with a Nano-ZS90 instrument (Malvern, UK) and TEM images were acquired using a JEOL JEM 2100F instrument at an accelerating voltage of 200 kV. Size distribution of AuNPs was calculated using ImageJ software. ELISA was performed using a microplate reader (Synergy H1 Hybrid Multi-Mode Reader, BioTek, Winooski, VT, USA) equipped with a 96-well plate. Raman spectra were measured with a Renishaw inVia Raman microscope system (Renishaw, New Mills, UK). A He-Ne laser at 633 nm was used as the excitation source with a power of 20 mW. The Rayleigh line was removed using an edge filter located in the collection path. Raman scattering signal was collected using a charge-coupled device (CCD) camera at a spectral resolution of 1 cm^−1^. BoNT immunocomplexes from microtube were transferred to a capillary tube, and their Raman scattering signals were measured by focusing a laser spot on the tube using a 20× objective lens. Baseline correction for each Raman spectrum was performed using Renishaw WIRE 4.0 software.

## 3. Results and Discussion

**Figure 1:**
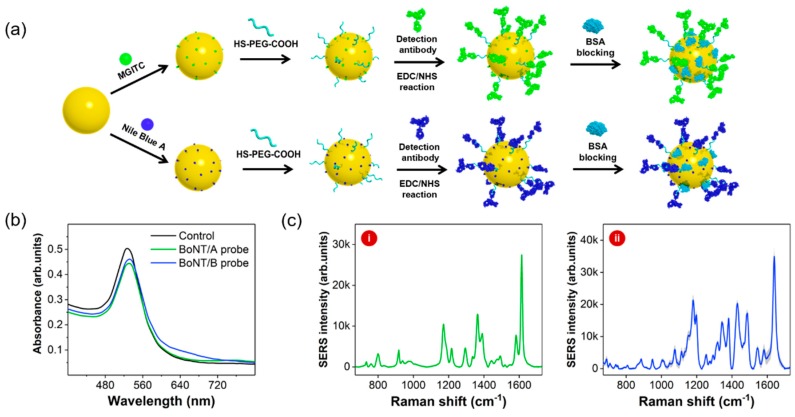
(**a**) Sequential process for the synthesis of two different types of BoNT SERS nanotags. (**b**) UV/Vis spectra for AuNPs (black), BoNT/A (green), and BoNT/B (blue) SERS nanotags. (**c**) Raman spectra for BoNT/A (i) and BoNT/B (ii) SERS nanotags.

Figure S1a shows the TEM image of AuNPs synthesized with the seeded-growth method. The average diameter was estimated to be 45 ± 5 nm. Figure S1b shows the size distribution of AuNPs, as determined with DLS measurements. The error bars indicate standard deviations from three measurements. Figure 1 demonstrates the process for the preparation of two different types of detection antibody-conjugated SERS nanotags. In Figure 1a, two Raman reporter molecules (MGITC and NBA) were adsorbed on the surfaces of AuNPs. BoNT antibodies (types A and B) were subsequently immobilized on the surfaces of AuNPs using HS-PEG-COOH. Optimization of the amount of HS-PEG-COOH was important to retain the stability of SERS nanotags. To determine the optimum concentration, six different concentrations of HS-PEG-COOH were tested with the colloidal solution containing AuNPs. After centrifugation, the pellet was re-suspended in PBS and the aggregation properties were investigated.

**Figure 2:**
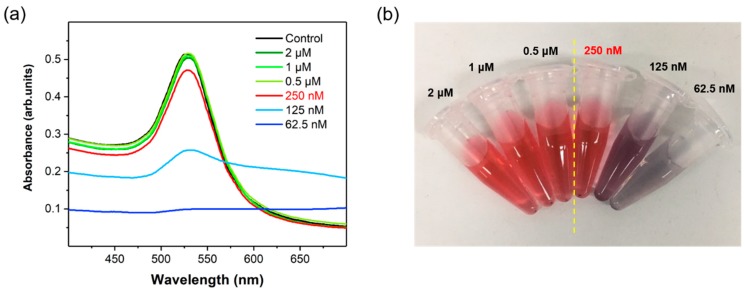
(**a**) UV/Vis spectra of PEGylated AuNPs for various concentrations of HS-PEG-COOH in the range of 63 nM to 2.0 μM. (**b**) Corresponding image of PEGylated AuNPs.

Figure 2a shows the UV/Vis spectra of PEGylated AuNPs for various concentrations of HS-PEG-COOH in the range of 63 nM to 2.0 μM. Figure 2b shows the corresponding images of PEGylated AuNPs. As shown in these figures, aggregation was observed from a concentration of 250 nM HS-PEG-COOH along with a change in the color of the solution to deep purple at concentrations lower than 125 nM. In the presence of low concentration of HS-PEG-COOH, the negative charge of citrate ions on the surface of AuNPs is neutralized by the salts in PBS. Therefore, AuNPs aggregate; however, higher concentrations of HS-PEG-COOH stabilize the surface of AuNPs. Hence, the optimum concentration of HS-PEG-COOH for the stabilization of SERS nanotags was determined to be 0.50 μM. Detection BoNT/A and BoNT/B antibodies were conjugated on AuNPs using EDC/NHS coupling reactions. No significant change of Raman signal intensities was observed after antibody conjugation. Capture antibody-conjugated magnetic beads were prepared with a similar method. Carboxylic acid-functionalized magnetic beads were activated with EDN/NHS and immobilized with capture BoNT antibodies. UV/Vis spectral data in Figure 1b show that the surface plasmon bands for both BoNT/A and BoNT/B were slightly shifted from 527 nm to 531 nm upon immobilization of the corresponding antibodies on the surfaces. These spectral changes confirm that both BoNT antibodies were successfully immobilized on the surfaces of AuNPs. Raman spectra of BoNT/A (i) and BoNT/B (ii) SERS nanotags in Figure 1c also demonstrate the successful adsorption of the Raman reporter molecules on the surfaces of AuNPs.

Figure S2a shows the sequential process for the preparation of capture BoNT antibody-conjugated magnetic beads. Carboxylic acid-functionalized magnetic beads were used for the fabrication of capture substrates. The surfaces of magnetic beads were activated with the NHS/EDC coupling reaction, and then immobilized with BoNT antibodies. The remaining sites were treated with BSA to prevent any nonspecific binding. The conjugation was determined with the enzyme-catalyzed reactions. Anti-mouse IgG HRP conjugates were incubated with bare and antibody-conjugated magnetic beads. The addition of TMB and TMB stop buffer to each well resulted in a change in the color of antibody-conjugated magnetic beads from colorless to yellow. Enzyme immunoassays frequently incorporate the use of HRP as the enzyme label. This enzyme usually catalyzes the oxidation of a chromogen which can be quantified after termination of the enzyme reaction. A chromogen widely used for this purpose is TMB. The absorbance at 450 nm was measured to quantify the target BoNT toxins. However, no color change was detected for bare magnetic beads. Figure S2b demonstrates the difference in the relative absorption intensities at 450 nm for BoNT/A (i) and BoNT/B (ii) antibody-conjugated magnetic beads. Herein, the histogram for control indicates the absorption intensity of bare magnetic beads.

**Figure 3:**
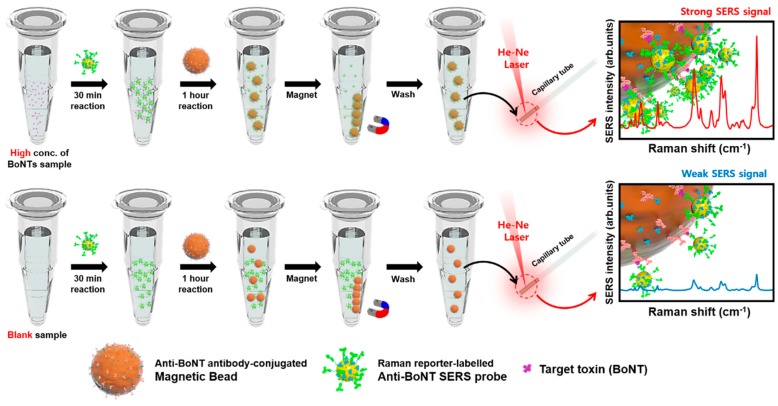
Schematic illustration of the sequential SERS-based immunoassay process, including mixing of BoNT toxin and BoNT antibody-conjugated magnetic beads, addition of BoNT antibody-conjugated SERS nanotags, separation of magnetic immunocomplexes, and SERS detection.

Figure 3 shows the schematic illustration of the sequential process of SERS-based immunoassays using magnetic beads and SERS nanotags. Target BoNT toxins were mixed with detection antibody-conjugated SERS nanotags in a microtube, and then captured by the capture antibody-conjugated magnetic beads through antigen-antibody immunoreactions. We used a magnetic bar to collect magnetic sandwich immunocomplexes on the wall of the microtube, and then washed the supernatant solution with PBST three times using a micropipette. The magnetic immunocomplexes were re-dispersed in PBS and the solution was transferred into a capillary tube for the analysis of Raman signals. In the presence of target BoNT toxins, strong Raman signals were observed; however, Raman signals were relatively weak in the absence of BoNT toxins.

**Figure 4:**
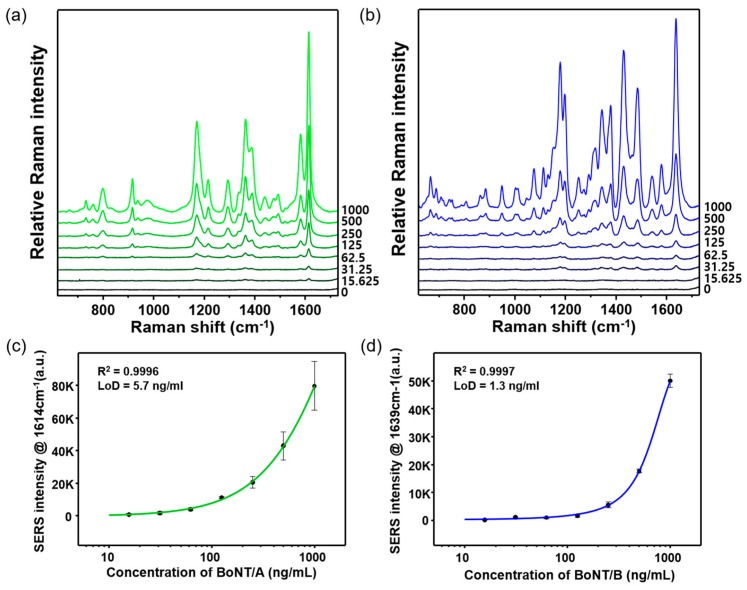
Raman spectra of various concentrations of (**a**) BoNT/A and (**b**) BoNT/B. Concentration range for BoNT/A and BoNT/B was 0 to 1000 ng/mL. Corresponding calibration curves for (**c**) BoNT/A and (**d**) BoNT/B toxins. The error bars indicate standard deviations from three measurements.

In the present work, the strongest Raman peak intensities at 1614 cm^−1^ (MGITC) and 1639 cm^−1^ (NBA) were used for the quantitative evaluation of BoNT/A and BoNT/B toxins, respectively. Figure 4a,b shows the Raman spectra for various concentrations of BoNT/A and BoNT/B, respectively. Their concentrations varied from 0 ng/mL to 1.0 μg/mLml. In the absence of BoNTs, weak Raman signals were observed owing to the formation of few immunocomplexes in the microtube; hence, most SERS nanotags in the supernatant solution were removed during washing. An increase in BoNT concentration, however, led to the formation of more immunocomplexes, as evident from the corresponding increase in Raman signal intensities. Figure 4c,d demonstrates the corresponding calibration curves for BoNT/A and BoNT/B, as constructed from the intensity variations at 1614 cm^−1^ (MGITC) and 1639 cm^−1^ (NBA), respectively. The error bars indicate standard deviations from the measurements for three immunocomplex replicas. The LODs, as determined with the SERS-based immunoassay method, were 5.7 ng/mL (R^2^ = 0.999) and 1.3 ng/mL (R^2^ = 0.999) for BoNT/A and BoNT/B, respectively.

**Figure 5:**
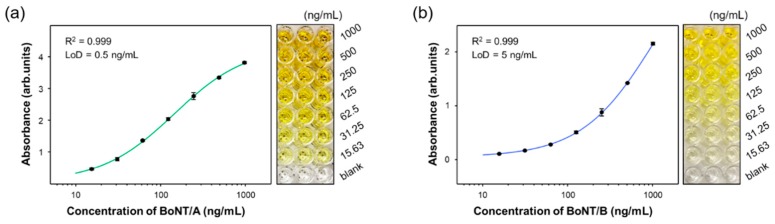
ELISA analysis using a 96-well plate and corresponding calibration curves for various concentrations of (**a**) BoNT/A and (**b**) BoNT/B toxins. The error bars indicate standard deviations from three measurements.

ELISAs were also performed for both BoNT toxins to evaluate the SERS-based immunoassays using magnetic beads and SERS nanotags. Figure 5 shows the ELISA standard curves and images of 96-well plates for different concentrations of BoNT/A (a) and BoNT/B (b). The dynamic ranges for both BoNT toxins varied from 0 ng/mL to 1.0 μg/mL. The increase in BoNT concentration led to a change in the color from colorless to deep yellow. The related LODs determined by ELISA were 0.5 ng/mL (R^2^ = 0.999) and 5.0 ng/mL (R^2^ = 0.999) for BoNT/A and BoNT/B, respectively.

## 4. Conclusions

In the present study, we developed a SERS immunoassay technique for the rapid and sensitive detection of BoNT/A and BoNT/B using SERS nanotags and magnetic beads. Two different types of detection antibody-conjugated SERS nanotags, labeled with MGITC and NBA, and capture antibody-conjugated magnetic beads were fabricated for the dual detection of BoNT/A and BoNT/B. Total assay time was less than 2 h, including immunoreaction, washing, and detection, and the sample volume needed was lower than that for ELISA.

## Supplementary Materials

The following are available online at https://www.mdpi.com/1424-8220/19/19/4081/s1, Figure S1: TEM image and size distribution of the synthesized AuNPs; Figure S2: Preparation of an antibody-conjugated magnetic bead and identification of antibody conjugations for BoNT/A and BoNT/B.
