# Supplementary material for: SERS-Based Immunoassays for the Detection of Botulinum Toxins A and B Using Magnetic Beads
Source: Sensors (Basel). 2019 Sep 21;19(19):4081. doi: 10.3390/s19194081 (PMC6806190; doi:10.3390/s19194081)
Supplement: Supplementary file 1 [file sensors-19-04081-s001.pdf]

Supplementary Materials

SERS-based Immunoassays for the Detection of Botulinum Toxins A and B Using Magnetic Beads

Kihyun Kim [1], Namhyun Choi [2], Jun Ho Jeon [3], Gi-eun Rhie [3] and Jaebum Choo [1,*]

[1] Department of Chemistry, Chung-Ang University, Seoul 06974, Korea; sadtiger92@naver.com
[2] Department of Bionano Technology, Hanyang University, Ansan 426-791, Korea; choi.namhyun@gmail.com
[3] Division of High-risk Pathogens, Laboratory Control of Infectious Diseases, Korea Centers for Disease Control and Prevention, Chungju 28159, Korea; jhjeon78@korea.kr; gerhie@korea.kr
[*] Correspondence: jbchoo@cau.ac.kr; Tel.: +82-2-820-5801

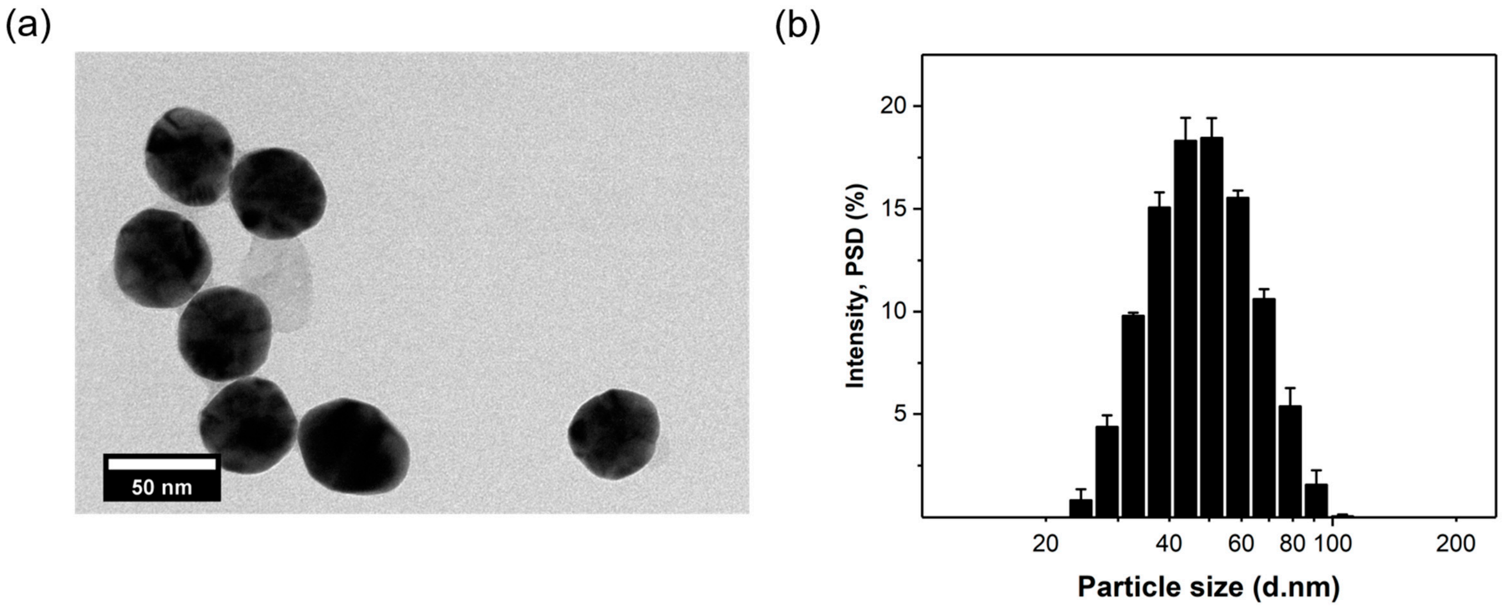

Figure S1. (**a**) TEM image of synthesized AuNPs. (**b**) Size distribution of AuNPs obtained from DLS measurements.

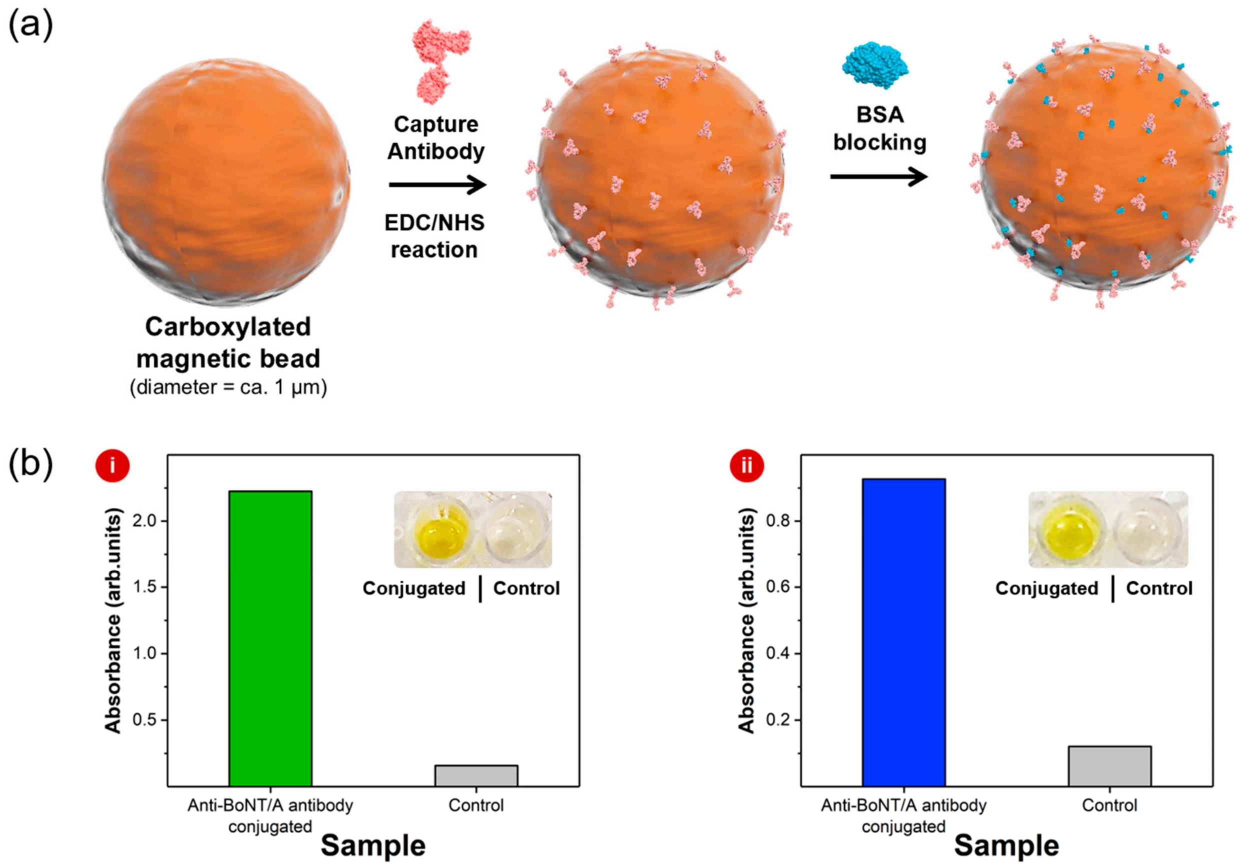

Figure S2. (**a**) Sequential process for preparation of capture BoNT antibody-conjugated magnetic beads. (**b**) Relative absorption intensities at 450 nm for BoNT/A (i) and BoNT/B (ii) antibody-conjugated magnetic beads. Controls indicate the histograms of the absorption intensity values for bare magnetic beads.
